# Food-drug interactions of novel drugs used in cardiologic and diabetic patients – a PRISMA scoping review

**DOI:** 10.3389/fphar.2026.1786007

**Published:** 2026-04-27

**Authors:** Iga Pawłowska, Karolina Kuźbicka, Anna Kowalczys, Leszek Pawłowski, Ivan Kocić

**Affiliations:** 1 Department of Pharmacology, Medical University of Gdańsk, Gdańsk, Poland; 2 First Department of Cardiology, Medical University of Gdańsk, Gdańsk, Poland; 3 Department of Palliative Medicine, Medical University of Gdańsk, Gdańsk, Poland

**Keywords:** bioavailability, bioequivalence, drug adsorption, food effect, food–drug interactions

## Abstract

**Background:**

Food–drug interactions are common in orally administered therapies. In order to achieve optimal therapeutic efficacy and safety, it is important for patients to understand the most appropriate way to take their medications in accordance with their diet.

**Objectives:**

To evaluate the impact of food on the oral bioavailability of novel cardiovascular and antidiabetic drugs, including sacubitril/valsartan, direct oral anticoagulants, sodium-glucose cotransporter-2 inhibitors, semaglutide, vericiguat, pitavastatin, and bempedoic acid.

**Methods:**

PubMed, Scopus, and Cochrane Library databases were searched from inception to May 2024, following the guidelines of the PRISMA-ScR (Preferred Reporting Items for Systematic Reviews and Meta-Analyses Extension for Scoping Reviews) statement. The search strategy employed keywords including ‘food drug interactions’, ‘food effect’, ‘bioavailability’, and ‘bioequivalence’, combined with the names of the investigated drugs. Eligible publications comprised clinical pharmacokinetic studies and randomized clinical trials evaluating the effect of food on drug bioavailability.

**Results:**

A total of 36 publications met the inclusion criteria. For most drugs, food was found to reduce the maximum concentration and delay the rate of absorption of the active substance without significantly affecting overall drug exposure. Therefore, these drugs can generally be administered regardless of meals. Semaglutide is recommended to be administered on an empty stomach, and pitavastatin demonstrated higher bioavailability in the fasted state. In contrast, vericiguat and higher doses of rivaroxaban are better absorbed when taken with food. For patients with swallowing difficulties, rivaroxaban, apixaban, and edoxaban may be crushed and administered either via a nasogastric tube or orally mixed with applesauce.

**Conclusion:**

For a substantial proportion of novel cardiovascular and antidiabetic therapies, dosing without strict regard to meals is supported by available evidence. This level of flexibility may facilitate patient adherence and contribute to improved therapeutic outcomes in clinical practice.

## 1 Introduction

### 1.1 Rationale

**FIGURE 1:**
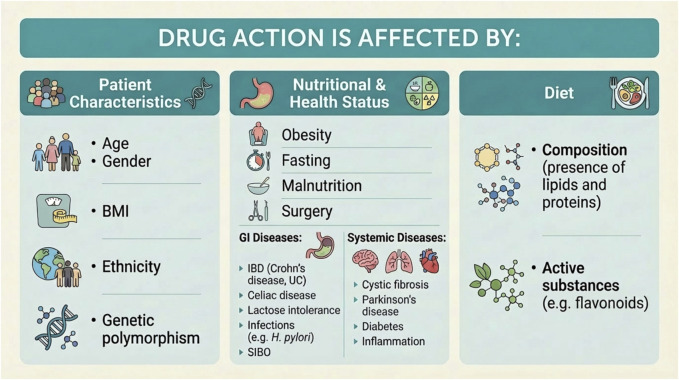
Factors affecting drug action (BMI–body mass index, IBD–inflammatory bowel disease, UC–ulcerative colitis, SIBO–small-intestinal bacterial overgrowth). The Figure 1 was made with the help App GPAI (only for creation graphics).

Drug action within the body is affected by a multitude of interrelated factors. These can be divided into three main categories: patient characteristics, nutritional and health status, and diet. Figure 1 provides further details of these variables. Patient characteristics are defined by an individual’s age, sex, body mass index (BMI), ethnicity, and genetic polymorphisms. Nutritional and health status is affected by patient obesity, fasting, malnutrition, surgery, and comorbidities. The third category, diet, relates to the type and composition of food consumed by patients (D’Alessandro et al., 2022; de Jong et al., 2020; Meyer, 2000; Monés et al., 1993; Nimmo and Peacock, 1988; Shenfield, 2004; Zarezadeh et al., 2020). The concomitant ingestion of food or beverages can significantly alter the properties of orally administered drugs–a phenomenon referred to as food–drug interactions (FDIs). The term “food effect” forms a subset of FDIs and primarily relates to the impact on pharmacokinetic parameters such as the area under the concentration–time curve (AUC), maximum plasma concentration (C_max_), time to maximum plasma concentration (T_max_) and overall bioavailability. In the context of drug absorption in under fed versus fasted conditions, “food effect” is commonly used to describe absorption-related FDIs (Cheng and Wong, 2020; Deng et al., 2017).

### 1.2 Patient-specific factors influencing drug action

Gender can significantly modulate food–drug interactions through its effect on drug metabolism (notably via CYP3A4 activity), gastric emptying, and hormonal influences. In women, reduced gastrointestinal motility may alter the rate and extent of drug absorption, whereas men typically exhibit less interindividual variability (Bennink et al., 1998; Chetty et al., 2012; Monés et al., 1993). When considering hormonal influences, a female’s menstrual cycle may also have an impact on the metabolism of drugs (Chetty et al., 2012).

Age plays a critical role, as age-related changes in gastrointestinal physiology, hepatic and renal function, body composition, and enzymatic activity can markedly influence both the pharmacokinetic and pharmacodynamic consequences of food–drug interactions (Batchelor, 2015; Ngcobo, 2025).

Body weight may affect the activity of hepatic enzymes on drug biotransformation. Obesity may decrease the activity of CYP3A4/5, CYP1A2 and CYP2C9 and increase the activity of CYP2E1, whereas on the contrary weight loss may increase the activity of CYP3A4. Interestingly, evidence indicates that the activity of CYP1A2 is increased by a high protein diet and/or fasting, however is decreased by diet high in carbohydrates (Zarezadeh et al., 2020).

The aforementioned factors are generally difficult or impossible to modify, and their presence tends to remain constant. In contrast, dietary factors merit consideration, as they are modifiable and, when patients are aware of them, their negative effects can be more readily minimized or avoided, while beneficial effects can be achieved.

### 1.3 Food-drug interactions

Oral administration remains the most prevalent route of drug delivery, due to its convenience, patient compliance, widespread acceptance, and cost-effectiveness (Kim and De Jesus, 2026). Food can influence drug action in various ways. On the one hand, it may enhance a drug’s effect by increasing its bioavailability, whereas on the other hand, it may reduce plasma drug concentrations, potentially leading to therapeutic failure. Additionally, food–drug interactions may also cause or intensify adverse drug reactions (ADR’s). These interactions can involve changes in the pharmacokinetic or pharmacodynamic parameters of the drug (Wang et al., 2025). A review aimed at identifying herb and dietary supplement interactions with food indicated that 42.3% of reported ADRs impacted on pharmacokinetic mechanisms, 40.1% on pharmacodynamics and 8.5% on both (Tsai et al., 2012).

Pharmacokinetic interactions alter drug absorption, distribution, metabolism and elimination, which may consequently affect the bioavailability of the active substance. Among these, the absorption phase is particularly sensitive to interactions with food, as the physiochemical and physiological conditions within the gastrointestinal tract can be markedly altered following a meal. Food modifies luminal conditions such as pH, fluid volume, and motility, all of which play a key role in drug absorption. For example, the availability and volume of luminal fluids are particularly critical for drugs with poor aqueous solubility (Schiller et al., 2005). Certain foods can increase (i.e., lettuce and rhubarb meals) (Wilkinson-Smith et al., 2018) or decrease (whole meal bread) (Marciani et al., 2013) luminal fluid volume, directly impacting drug solubility and, consequently, absorption. Another important factor is the change in gastric and intestinal motility. Food intake clearly affects small intestinal transit time, which in turn, can alter drug bioavailability (Fadda et al., 2009). In addition, food-induced changes in luminal pH may influence the ionization of drugs, thereby affecting their solubility and absorption (Koziolek et al., 2015). Gastric pH varies between the fed and fasted states, because of the buffering effect of a meal (Simonian et al., 2005). Postprandial increases in pH are mainly seen in the stomach and can affect the absorption of ionizable drugs. For weakly acidic drugs, elevated postprandial pH can accelerate absorption by enhancing the dissolution rate from a solid formulation. However, at the same time this can also lower the amount of unionized drug which decreases the bioavailability of the active substance. For weakly basic drugs, higher pH’s can lead to a larger percentage of drugs in their unionized form, expediting their absorption (Deng et al., 2017).

The composition of an individual’s diet impacts on pharmacokinetic variability through the modulation of gut microbiota - which are increasingly being recognized for their role in drug metabolism (Sousa et al., 2014). Diet composition (e.g., animal-vs. plant-based diets) can shift the intestinal microbial environment and thereby influence drug absorption and bioavailability (David et al., 2014). Drugs can utilize the same intestinal transport mechanisms as nutrients, resulting in potential competition. A number of transporters located in intestinal epithelial cells or in hepatocytes are responsible for mediating the absorption of both food components and pharmaceuticals, which contributes to the incidence of food–drug interactions (Nakanishi and Tamai, 2015). Its presence as well as the type of food ingested has a significant impact on oral absorption. Lipids are a constituent of food known for interfering in drug adsorption. They slow down gastric motility and delay gastric emptying, which can increase the absorption of lipid soluble drugs. Digested lipids may also enhance the transport of poorly water-soluble drugs through the biologic membranes, thereby increasing absorption (Charman et al., 1997). Protein-rich foods can influence oral drug absorption in complex ways. On one hand, they can enhance absorption by increasing postprandial splanchnic blood flow, on the other, once digested into small peptides and amino acids, they can compete with drugs for uptake transporters, thereby inhibiting transporter-mediated absorption (Deng et al., 2017).

Beyond absorption, food can also affect the distribution of drugs. Highly lipophilic drugs, such as steroidal hormones, are better absorbed and distributed more rapidly when co-administered with a lipid-rich meal (White et al., 2009). The presence of food can also affect drug metabolism and elimination by modulating the activity of cytochrome P450 enzymes (Won et al., 2012). The most well-known example of these interactions is the inhibition of the metabolism of CYP3A substrates by grapefruit juice (Edwards et al., 1999).

FDI’s can also be pharmacodynamic in nature and they may directly interfere with drug action or trigger adverse reactions (D’Alessandro et al., 2022). They are usually caused when a specific component of the food, nutrient and/or dietary supplement disrupts the pharmacological effect of the drug (Koziolek et al., 2019). These components include, among others, tyramine interacting with antidepressants, kava, astragallus, licorise, saw palmetto, garlic, magnesium, potassium and calcium (Tsai et al., 2012). Pharmacodynamic interactions are particularly relevant when considering drugs with narrow therapeutic windows, such as warfarin. Evidence highlights that certain foods rich in vitamin K including spinach, sushi, green tea, avocado, broccoli, parsley and Japanese food can reduce the anticoagulant effects of warfarin (Tan and Lee, 2021). When considering ADR’s, the risk of thrombocytopenia while using anticancer drugs may be potentiated by the concomitant usage of white willow extracts (Salix Alba), which contain salicylates (Clauson et al., 2005).

### 1.4 Diseases that may affect food-drug interactions (FDIs)

Although current guidelines are largely based on studies conducted in healthy subjects, most patients taking medicines may suffer from various diseases that alter the physiological parameters of the gastrointestinal tract (Stillhart et al., 2020). These parameters include gastric emptying rate, pH, transit times, intestinal surface area, epithelial permeability, intestinal enzyme activity, transporter expression, luminal fluids, and the intestinal microbiota (Bai et al., 2016; Stillhart et al., 2020). It is well established that many systemic and gastrointestinal diseases modify these factors and, consequently, affect drug absorption. Concomitant food intake can additionally modify these already altered physiological conditions, producing further variability in drug dissolution, solubilization, and uptake (Effinger et al., 2019; Hatton et al., 2019).

Inflammatory bowel diseases (IBD), primarily Crohn’s disease and ulcerative colitis, represent the most prevalent pathological conditions of the gastrointestinal (GI) tract. In Crohn’s disease, gastric emptying is often delayed, particularly in the fed state, while small intestinal transit time tends to be accelerated (noting the potential for considerable interindividual variability) (Effinger et al., 2019). In ulcerative colitis, chronic diarrhoea and rectal bleeding are frequent clinical features. As a result, patients typically exhibit prolonged colonic transit compared with healthy individuals; however, alterations in gastric residence time are generally minimal (Effinger et al., 2019).

IBD is characterized by inflammation affecting the full thickness of the bowel wall, which has significant implications for drug absorption. On one hand, the absorptive surface area is reduced; on the other, intestinal permeability is increased–a phenomenon commonly described as “leaky gut syndrome.” Functional disturbances such as chronic diarrhoea or constipation can further modify the pharmacokinetics of orally administered drugs. Structural complications, including segmental narrowing and potential obstruction, may also impair the movement of luminal contents and pharmaceutical formulations. Consequently, drug absorption in patients with IBD often deviates from that in the healthy population, although the magnitude of these changes remains unpredictable due to the heterogeneity of the disease (Stillhart et al., 2020).

Research on drug absorption in celiac disease is limited, outdated, and inconsistent, with several potential FDI’s identified; however more robust studies are needed to draw clear conclusions (Stillhart et al., 2020). One review observed a decrease in the absorption of paracetamol, levothyroxine and some antibiotics, and an increased level of propranolol absorption (Tran et al., 2013). In patients with lactose intolerance, levothyroxine absorption may already be reduced due to maldigestion, often resulting in the need for higher doses to achieve and maintain euthyroid status. Additionally, since many levothyroxine formulations contain lactose as an excipient, their use in these patients may exacerbate intolerance-related symptoms and further compromise absorption. Therefore, in cases where there is an unexplained poor response to levothyroxine therapy–characterized by persistent elevation of TSH despite confirmed adherence–lactose intolerance should be considered as a potential contributing factor (Asik et al., 2014).

Gastrointestinal tract infections may also influence the absorption of drugs. *Helicobacter pylori* infection can increase gastric pH (up to 4–7) due to the production of urease by the bacteria, which hydrolyzes urea to ammonia and carbon dioxide. The ammonia acts as a buffer, reducing stomach acidity. Under these conditions, weakly acidic drugs (pKa 3–5) show reduced absorption, while weakly basic drugs (pKa 7–9) may have improved bioavailability (Mitra and Kesisoglou, 2013; Sachs et al., 2009). Therefore, *H. pylori* infection alters the absorption of various drugs (e.g., levodopa, levothyroxine, delavirdine), which require a certain pH for optimal absorption (Lahner et al., 2014).

Small-intestinal bacterial overgrowth (SIBO) can impair the intestinal absorption of some vitamins and minerals (notably vitamin B12 B1, B3) due to the underlying pathophysiological mechanisms of the condition including altered intestinal motility, maldigestion, and symptoms such as chronic watery or fatty diarrhoea, bloating, abdominal pain and constipation. However, the condition elevates levels of vitamin K and folate due to bacterial synthesis (Roszkowska et al., 2024).

Systemic diseases that affect the GI tract can also impact on drugs’ bioavailability. In cystic fibrosis, bicarbonate secretion is impaired, causing a pH increase and a decrease of pancreatic enzyme release. As a result, drug absorption is highly variable and often reduced due to pancreatic insufficiency, fat malabsorption, altered GI transit and pH, abnormal mucus, and microbial dysbiosis, necessitating individualized dosing or alternative delivery routes (Hatton et al., 2019; Stillhart et al., 2020). In this condition, food can have a significant impact on drug absorption–fatty meals may enhance the uptake of lipophilic drugs if pancreatic enzymes are supplemented.

Gastric motility and gastric emptying (which affects timing and extent of drug exposure) may be decreased in Parkinson’s disease, diabetes mellitus type-2 (T2DM), multiple sclerosis and hypothyroidism, resulting in lower drug liberation and delayed time to maximum plasma concentration (T_max_) (Hatton et al., 2019; Su et al., 2017). In T2DM, an observed increase in gastric and intestinal pH, reduced gastric mucosal blood flow, and gut microbiota dysbiosis impairs the dissolution, disintegration, and bioavailability of pH-sensitive drugs (Hatton et al., 2019). In Parkinson’s disease, drug absorption (oral levodopa) is often delayed and unpredictable due to reduced absorptive surface area and gut microbiome disturbances. Strategies to overcome these challenges include the use of liquid or duodenal/jejunal formulations and *H. pylori* eradication to improve bioavailability (Hatton et al., 2019).

During chronic inflammatory states, key drug-metabolizing enzymes and transporters are modulated, which can alter the pharmacokinetics of orally administered drugs. This inflammatory status may have implications on food-drug effects, further influencing absorption, metabolism, and systemic exposure. By integrating inflammatory markers with pharmacogenetic profiling and therapeutic drug monitoring, therapy can be personalized to optimize drug efficacy and minimize adverse effects. Understanding the combined impact of inflammation and food-drug interactions is therefore critical for predicting interindividual variability and ensuring safe and effective pharmacotherapy (Aitken and Morgan, 2020).

### 1.5 Biopharmaceutics Classification System (BCS) and Biopharmaceutics Drug Disposition Classification System (BDDCS)

Thirty years ago, Amidon et al. (1995) introduced the Biopharmaceutics Classification System (BCS) (Amidon et al., 1995). The BCS is a scientific framework developed to categorize orally administered drugs based on their aqueous solubility and intestinal permeability. It is primarily utilized to predict oral absorption potential and bioavailability to support regulatory decisions, such as the granting of biowaivers for *in vivo* bioequivalence studies. BCS is particularly informative for understanding how food intake, especially high-fat meals, may alter drug bioavailability. Each of the four BCS classes demonstrates distinct characteristics with respect to absorption and potential food effects:

- class I includes drugs with both high solubility and permeability (food usually does not have a significant effect on absorption);
- class II includes drugs with low solubility, and high permeability (high-fat food may enhance absorption by improving solubilization within the gastrointestinal tract);
- class III includes drugs with high solubility but poor permeability (absorption is present at the specific site i.e., proximal intestine, so sometimes food may delay absorption);
- class IV contains drugs with low solubility and permeability (generally unpredictable food effect, however, sometimes may be positive) (Wang et al., 2015).

Approximately a decade later, Wu and Benet proposed the Biopharmaceutics Drug Disposition Classification System (BDDCS) based on the major route of drug elimination and solubility. According to this system, all drugs are divided into four classes: (1) extensive metabolism and high solubility, (2) extensive metabolism and low solubility, (3) poor metabolism and high solubility, (4) poor metabolism and low solubility (Wu and Benet, 2005). Apart from predicting drug elimination, this system may also predict drug disposition in the body, drug-drug interaction and consequently food-drug interactions (Custodio et al., 2008). The authors of this thesis explained the food effect on intestinal transporters. For example,: high fat meals and lipidic excipients may inhibit transporters, therefore this form of food may increase the bioavailability of class 2 drugs (due to a decrease in drug extraction), however will have the opposite effect on class 3 drugs and minimal effect on class 1. The classification of BDDCS may predict absorption and bioavailability in high-fat meal for approximately 70% of drugs (Custodio et al., 2008).

### 1.6 Objectives

For oral drugs that have been in use for a while, both medical staff and patients are generally familiar with administration protocols. In contrast, attention needs to be directed toward newly introduced oral drugs, as guidelines for their administration under fasted and fed conditions are not often widely disseminated or well understood. This is particularly relevant for medications used by patients with cardiovascular diseases (CVD), such as angiotensin receptor neprilysin inhibitor (ARNI), vericiguat, direct oral anticoagulants (DOACs, which have been replacing vitamin K antagonists–VKAs), bempedoic acid and pitavastatin as well as drugs used in T2DM: oral incretin drugs or sodium-glucose cotransporter inhibitors (SGLT2 inhibitors), which are indicated for both conditions. However, none of the drugs analyzed in this review have a narrow therapeutic index and the drugs are considered as safe, the knowledge regarding the possible interactions may increase the therapeutic effectiveness and reduce the adverse drug reactions (Bhat et al., 2022; Boettcher et al., 2020; Clark, 2018; Laufs et al., 2019; Sadeq et al., 2023; Yang and Yang, 2024). For example, DOACs are preferred over traditional VKAs because they have a wider therapeutic window, fewer drug- or food-related interactions, and do not require routine monitoring. Nonetheless, their pharmacodynamic effects mean they may still carry substantial risk (Clark, 2018). Understanding FDIs can help healthcare providers to not only assess the bioavailability of drugs in individual patients but also determine the correct dosage of oral drugs in order to obtain the desired effect. The provision of education to patients about potential FDIs can improve adherence to medication regimens. Patients who understand these interactions are more likely to follow dietary recommendations and avoid foods that could interfere with their medication (Zaidi et al., 2021). Improving knowledge around FDIs is important in order to be able to achieve optimal therapeutic effects, minimize the incidence of side effects, and ensure patient safety.

The objective of this scoping review is to map and characterize the available clinical evidence on the effects of food intake on the oral bioavailability of recently introduced cardiovascular and antidiabetic pharmacotherapies, specifically sacubitril/valsartan, vericiguat, rivaroxaban, edoxaban, apixaban, dabigatran, empagliflozin, dapagliflozin, canagliflozin, ertugliflozin, bempedoic acid, pitavastatin, and semaglutide. Furthermore, the study aims to classify these agents according to the Biopharmaceutics Classification System (BCS) and the Biopharmaceutics Drug Disposition Classification System (BDDCS) to facilitate a more rigorous interpretation of food-effect outcomes on drug absorption. By integrating pharmacokinetic outcomes with Biopharmaceutics classification frameworks, this scoping review seeks to provide an organized overview of existing evidence to support the interpretation of food–drug interaction data and inform clinical considerations regarding drug administration in relation to food intake.

## 2 Materials and methods

### 2.1 Study design

A scoping review of the literature was performed using comprehensive search strategies. The scoping review was reported in accordance with the Preferred Reporting Items for Systematic Reviews and Meta-Analyses extension for Scoping Reviews (PRISMA-ScR) to ensure methodological transparency and reproducibility (Tricco et al., 2018; Veroniki et al., 2025). The review methodology followed the Population-Concept-Context (PCC) framework recommended for scoping reviews. The population included healthy volunteers and/or patients receiving novel cardiologic or antidiabetic drugs. The concept of interest was the impact of food intake on pharmacokinetic and/or pharmacodynamic parameters of these drugs. The context included clinical studies evaluating the effect of food on the bioavailability or pharmacokinetic profile of the investigated medications. The methodological approach was guided by the framework for scoping reviews proposed by Hillary Arksey and Lisa O'Malley and further refined by Danielle Levac and colleagues (Arksey and O’Malley, 2005; Levac et al., 2010).

### 2.2 Search strategy

Three electronic databases: PubMed, Scopus, and the Cochrane Library were searched from inception until 31 May 2024 and was limited to articles published in English. The search aimed to identify clinical studies evaluating the influence of food intake on the pharmacokinetics and/or pharmacodynamics of selected novel cardiologic and antidiabetic drugs.

The search strategy combined Medical Subject Headings (MeSH) and free-text keywords related to food–drug interactions and pharmacokinetic parameters. The primary MeSH terms included ‘Food-Drug Interactions’ and ‘Biological Availability’. In addition, free-text keywords such as ‘Food Effect’, ‘Bioavailability’ and ‘Bioequivalence’ were used to increase the sensitivity of the search.

These terms were combined with the names of the investigated drugs using Boolean operator AND. Filters for Clinical Trial (CT) and Randomized Controlled Trial (RCT) publication types were applied to focus the search on primary clinical studies assessing the influence of food intake on pharmacokinetic and pharmacodynamic outcomes. The search was conducted individually for each investigated drug. The search strategy was repeated separately for each investigated drug to ensure comprehensive identification of relevant clinical studies evaluating food–drug interactions.

### 2.3 Eligibility criteria

Randomized Controlled Trials (RCT) and Clinical Trials (CT) were included that investigated the influence of food intake (fed versus fasted conditions) on the pharmacokinetic parameters (AUC, C_max_, T_max_, and bioequivalence measures) and/or pharmacodynamic outcomes of newly introduced cardiologic and antidiabetic drugs. Studies involving both healthy volunteers and/or patients receiving these medications were considered eligible. Only studies reporting quantitative pharmacokinetic or pharmacodynamic outcomes were considered eligible.

The scope of the review included the following pharmacologic agents: sacubitril/valsartan, vericiguat, rivaroxaban, edoxaban, apixaban, dabigatran, empagliflozin, dapagliflozin, canagliflozin, ertugliflozin, bempedoic acid, pitavastatin, and semaglutide.

Studies evaluating the impact of food intake in under fed versus fasted conditions, including reporting on standardized or high-fat meals, were included. Reviews, systematic reviews, case reports, and clinical case studies were excluded in order to focus on primary clinical evidence.

### 2.4 Study selection

All records identified through the database searches were screened independently by two reviewers based on titles and abstracts, followed by full-text assessments where necessary. Potentially relevant studies were subsequently assessed through full-text review to determine eligibility. Any discrepancies in study selection were resolved through discussion and consensus. In addition, reference lists of included articles were manually screened to identify relevant studies not captured in the initial electronic search. The study selection process is summarized using a PRISMA-ScR flow diagram, detailing the number of records identified, screened, excluded, and included in the final scoping review (Figure 1).

### 2.5 Data charting process

Data from included studies were extracted using a predefined data charting form developed by the review team. Extracted information included: author and year of publication, study design, study population (healthy volunteers or patients), number of participants, drug investigated, fed and fasted conditions (including meal type when reported), pharmacokinetic and/or pharmacodynamic parameters assessed, and key findings related to food–drug interactions. In addition, food-related administration recommendations were verified using the prescribing information for the originator products approved by the U.S. Food and Drug Administration (FDA) and provided in the leaflet of the original product.

### 2.6 Synthesis of results

The extracted data were synthesized descriptively and presented in narrative and descriptive tabular formats. Results were grouped according to pharmacological class and individual drugs, with particular emphasis on the magnitude and clinical relevance of food-related effects on pharmacokinetic and pharmacodynamic parameters. No formal assessment of methodological quality or risk of bias of the included studies was conducted, as this is not a mandatory component of scoping reviews.

### 2.7 Establishing BCS and BDDCS classes

AI-assisted tools were used to facilitate the identification of relevant literature for determining the BCS and BDDCS classifications presented in Table 1. The literature search was conducted independently by three researchers. All retrieved sources were subsequently reviewed and verified by the authors, and the final classifications were assigned based only on the validated literature evidence.

**TABLE 1:** Characteristics of the analyzed medicines based on selected articles, BCS and BDDCS classification and recommendation regarding their proper usage (based on literature, drug labels, Food and Drug Administration information, European Medicines Agency, DrugBank, DrugCentral); (AUC–area under the curve, ARNI–angiotensin receptor neprilysin inhibitor, BCS–Biopharmaceutics Classification System, BDDCS - the Biopharmaceutics Drug Disposition Classification System, BMI–body mass index, C_max_–maximum concentration, DOAC–direct oral anticoagulants, FDC–fixed-dose combination, GI–gastrointestinal, GLP-1 – glucagon-like peptide, sGC–soluble guanylate cyclase, T_max_–time to maximum plasma concentration).

No	Group of drugs	Drug (citation)	Study design	Study population/Sample size	Main findings	Class BCS	Class BDDCS	Main clinical findings and information provided in the leaflet of the original product and FDA
1	ARNI	Sacubitril/valsartan, LCZ696 (Akahori et al., 2017)	Randomized, placebo-controlled study assessing the pharmacokinetics, food effect and safety of single ascending oral doses of sacubitril	healthy male Japanese subjects, aged 20–45 years BMI = 17–28 kg/m^2^ N = 50	Food reduced AUC and C_max_ of sacubitril by 21% and 72% (significant value) and sacubitrilat by 8% and 27% (not significant). Because sacubitril is a prodrug and there is no impact of food on its active metabolite, this interaction is not clinically meaningful Food reduced AUC of valsartan by 40%, and C_max_ by 51%, respectively	Sacubitril I (depending on the solubility) (Ayalasomayajula et al., 2017) Valsartan IV (low solubility in acidic pH and high in pH 6.8, low permeability) (de Castro et al., 2020)	Sacubitril: 1 (high permeability, extensive metabolism) (Ayalasomayajula et al., 2017) Valsartan: 4 (poorly metabolised) (Bocci et al., 2022)	Administration of sacubitril/valsartan with food has no clinically significant effect on systemic exposures of sacubitril, its active metabolite, or valsartan Sacubitril/valsartan is used twice a day administered and can be taken with or without food
2	ARNI	Sacubitril/valsartan, LCZ696 (Ayalasomayajula et al., 2016)	An open-label, randomized, 3-period crossover study evaluated the food effect on the oral bioavailability of LCZ696	healthy subjects; N = 36	C_max_ of sacubitril was decreased after low and high-fat meals by 42%–54% and of sacubitrilat by 19%–28% and T_max_ was increased when the drug was taken with food. Food slightly decreased systemic exposure of sacubitril (16%) and did not affect AUC of its active metabolite. In case of valsartan, the C_max_ decreased by 40% when the drug was taken with food and AUC by 33% when the drug was taken with low-fat meal, but this parameter was not changed by high-fat meal			
3	sGC stimulator	Vericiguat (Boettcher et al., 2021)	Six phase I studies were assessing safety, pharmacokinetics, pharmacodynamics and food effect on bioavailability of vericiguat	European, Chinese, Japanese healthy males, aged 18–45 years (mean 27.1–38.5) with a BMI 21.2–25.2 kg/m^2^; N = 255	Administration of vericiguat in slow released tablets with food increased AUC by 19%, C_max_ by 9%, reduced pharmacokinetic variability, and prolonged T_max_ (from 1-1.5h–4 h) in comparison to the fasting conditions	II (lipophilic, low water solubility, high permeability) (Becker et al., 2022)	2 (extensive metabolism) (Trujillo et al., 2023)	Vericiguat should be taken with a meal to enhance its bioavailability and improve the pharmacokinetic stability of the drug. It is taken once daily at about the same time each day. If the patient has problems with swallowing the tablet can be crushed and taken with water
4	DOAC	Rivaroxaban (Stampfuss et al., 2013)	Clinical trial, six independent, single-dose, cross-over studies examined the influence of food on pharmacokinetics, safety and tolerability of rivaroxaban.	healthy White male subjects N = 126 (13-24 in each study)	Various doses of rivaroxaban were tested under fed and fasted conditions: doses ≤10 mg – high oral bioavailability (80%) in both conditions doses: 15mg, 20 mg – high oral bioavailability (80%) was achieved in fed conditions	II (low water solubility, high permeability) (Kushwah et al., 2021)	2 (affected by metabolism and transporters) (Kou et al., 2021)	Rivaroxaban taken in doses to 10 mg had high oral bioavailability independent of food, therefore can be taken regardless of a meal. For higher doses (15mg, 20 mg) food delayed the T_max_ and increased C_max_ and AUC. It is recommended to take higher doses with food. For patients with difficulty in swallowing rivaroxaban can be crushed and taken orally in applesauce suspension or in water via NG tube
5	DOAC	Rivaroxaban (Kubitza et al., 2006)	Randomized clinical trial including 4 randomized studies (rivaroxaban and drug interactions: antacids and ranitidine, 2 food interaction studies)	healthy male subjects aged 18–45 years, with a BMI 18–32 kg/m^2.^ In food interaction study: aged 19–41 years, mean BMI 26.0–26.3 kg/m^2^; N = 46 (22 in food interaction study)	In the fed state, T_max_ was delayed by 1.25, C_max_ and AUC were increased by 41% and 28%, respectively Type of diet (high-fat, high-calorie and a high-carbohydrate) does not significantly change pharmacokinetic parameters. Regarding pharmacodynamic parameters and clinical outcomes, rivaroxaban in comparison to baseline increased PT prolongation of 44% and 53% in fasted state and 53% and 83% in fed conditions. Also, in fed conditions time to maximum PT prolongation was delayed by 0.5–1.5			
6	DOAC	Rivaroxaban (Moore et al., 2014)	Randomized control trial measured bioavailability of whole tablet, crushed tablet administered orally in applesauce suspension and crushed tablet administered in water via nasogastric (NG) tube	healthy adults, male and female, Black and White subjects aged 24–53 years, with a BMI 18.4–29.7 kg/m^2^; N = 55 (44 included)	C_max_ and AUC ∞ of crushed orally taken tablets and whole tablets were within the 80%–125% bioequivalence limits. AUC ∞ of crushed NG applied tablets and whole tablets was also within bioequivalence limits and C_max_ in this case was only slightly below the 80% lower bioequivalence limit Moreover, rivaroxaban did not adsorb to the NG-tubing and that the crushed rivaroxaban tablet remained stable for up to 4 h in the suspensions tested	​	​	​
7	DOAC	Edoxaban (Liu et al., 2022)	Open-label, randomized, 2-sequence, 4-period, crossover study investigated the bioequivalence of orally administered edoxaban 60-mg tablets and the food effects on the pharmacokinetics	healthy male and female, Chinese subjects; fasting cohort: aged 19–39 years (mean: 27.2), BMI 20.8–27.5 kg/m^2^ (mean: 24.0)^.,^ fed cohort: aged 20–45 years (mean: 28.8), BMI 20.2–27.5 kg/m^2^ (mean: 24.7) N = 64, (fasting cohort: N = 32 fed cohort: N = 32)	C_max_, AUC_0-t_, and AUC_0-_ ∞ were 97.0%, 95.4%, and 96.1%, in the fasting condition, and 98.6%, 100.0%, and 99.8%, respectively, in the fed conditions	IV (low solubility, low permeability) (Parasrampuria and Truitt, 2015)	4 (more than 50% of the drug is eliminated mainly unchanged) (Hosey et al., 2015)	There were modest, but no clinically significant effects of food on the pharmacokinetics and pharmacodynamics parameters of edoxaban. Edoxaban can be administered without regard to food. Edoxaban can be taken as either a crushed tablet mixed in applesauce and taken orally or suspended in water and administered via an NG tube
8	DOAC	Edoxaban (Duchin et al., 2018)	Phase 1, open-label, randomized, crossover study exanimated the bioequivalence of crushed tablet administered orally in apple pure or mixed with water and administered via NG tube	healthy adults, male and female, White, Black and other subjects aged 18–60 years with a BMI 18–30 kg/m^2^ (mean 26.3) N = 30	The C_max_ of edoxaban used in solid dosage formulation (tablet), crushed tablet administered orally in apple pure and crushed tablet administered in water via NG tube were 293, 283 and 303 ng/mL respectively, AUC ∞ 2089, 1993, 2051 ngxh/mL, respectively. T_max_ for crushed tablets was 1 h, and in case of whole tablet 1.5 h			
9	DOAC	Edoxaban (Mendell et al., 2011)	Open-label, randomized, 2-period crossover study to assess a high-fat meal on pharmacokinetics parameters of edoxaban used orally	healthy Japanese and Caucasian male subjects aged 20-55, BMI 19–29 kg/m^2^, drug free, and in good health N = 32, (Japanese cohort: N = 16 Caucasian cohort: N = 16)	The pharmacokinetic parameters as AUC_(0-t)_ AUC_(0-_ ∞ ), and C_max_ in fed state were higher in comparison to fast conditions, (from 6% to 22%)	​	​	​
10	DOAC	Edoxaban (Chen et al., 2017)	Open-label, phase-I trial investigating pharmacokinetics and pharmacodynamics of postprandial doses of edoxaban	healthy male and female Chinese subjects aged 18–45 years; N = 12 (6 male and 6 females)	C _max_ and AUC of edoxaban in fed state were like those after the same dose in fasting condition, and T_max_ was about half an hour longer. However, the exposure of its active metabolite was lower vs. the fasting condition, what may suggest the involvement of food on the active metabolite formation			
11	DOAC	Apixaban (Frost et al., 2013)	2 studies 1. Randomized, double-blind, placebo-controlled study assessed apixaban safety, pharmacokinetics and pharmacodynamics under fasted conditions. 2. An open label, randomized, two treatment crossover study investigated apixaban pharmacokinetics in fasted and fed conditions	healthy, Black and White male and female subjects aged 18–45 years (mean: 33), BMI 18–30 kg/m^2^ (mean: 24.9) 1.N = 57 2.N = 21	AUC and C_max_ was not affected by administration of a standard high fat, high calorie meal. Only T_max_ was increased by 1 h. The pharmacodynamic parameters such as INR (International Normalized Ratio), aPTT (activated Partial Thromboplastin Time) was comparable after fed and fasted conditions	III (Lee et al., 2023)	1 (Bocci et al., 2022)	Apixaban exposure was not affected by the administration of a standard high fat, high calorie meal. Apixaban can be taken with food. The results support safe administration of apixaban in various crushed forms for patients who cannot swallow tablets
12	DOAC	Apixaban (Song et al., 2015)	Open-label, randomized, crossover 3 studies evaluated among others the relative bioavailability of apixaban solution administered orally; via NGT flushed with either 5% dextrose in water or with infant formula or nutritional supplement as well crushed tablets administered in dextrose solution via NGT.	healthy Black and White, male and female subjects, aged 21–45 years, with a BMI 19.3–29.6 kg/m^2^ 1. N = 14 2. N = 21 3. N = 21	The following relative bioavailability values of apixaban were 1. Oral solution - 105%, versus whole tablet 2. Oral solution in 5% dextrose via NGT - 96.7% 3. Oral solution in infant formula flush via NGT- 92.2% 4. Oral solution with nutritional supplement via NGT -81,3% 5. Crushed tablet via NGT – 95,1%versus oral solution	​	​	​
13	DOAC	Apixaban (Song et al., 2016)	Two studies 1. An open-label, randomized, crossover study assessed the bioavailability of apixaban tablets administered as solid form, crushed and administered in water or mixed with applesauce 2. A second open-label, randomized, crossover study assessed apixaban tablet administered in fed and fasted conditions	healthy Asian, male and female subjects, aged 21–43 years (mean 31.2), BMI 19.1–29.1 kg/m^2^ (mean 23.6) 1. N = 33 2. N = 22	When apixaban was administered as crushed tablets mixed with applesauce, relative bioavailability was 86,3% in comparison to standard administration (bioavailability of crushed tablet and administered in water was 103%). Also C_max_ and AUC ∞ were by 21% and 16% lower After a high calorie meal, apixaban C_max_ and AUC_0–_ ∞ were 15% and 20% lower, respectively, in comparison to fasted conditions. T_max_ was also prolong in this conditions			
14	DOAC	Dabigatran (Stangier et al., 2005)	An open-label, 3-way crossover study examining the pharmacokinetics parameters of dabigatran used orally in the fasted and fed state and coadministration of pantoprazole	healthy volunteers and patients undergoing hip replacement therapy, male and female, aged 36–90 years, with a mean BMI 26.7 kg/m^2^ N = 77 (N = 18 healthy volunteers, N = 59 patients undergoing hip replacement therapy)	Food did not change the extend of dabigatran etexilate absorption. Coadministration of pantoprazole decreased mean AUC_*0-*_ ∞ (from 904 to 705 h ng/mL).	II (low solubility, high permeability) (Chai et al., 2016)	4 (changed to active metabolite, low bioavailability and poor metabolized) (Pollack, 2015)	Coadministration of food resulted in a delay in absorption of dabigatran, however food had no effect on the extent of absorption of dabigatran, therefore it can be used either fasting or fed condition. The capsules should be swallowed as a whole and not opened because this may increase the risk of bleeding
15	DOAC	Dabigatran (Li et al., 2020b)	Open-label, single-centre, ran-domized four-period crossover study comparing pharmacokinetic parameters of original and generic dabigatran under fed and fasted conditions	healthy Chinese male and female subjects fasting cohort: aged 18–39 years (mean 23.5), BMI 19.9–25.6 kg/m^2^ (mean 22.4); fed cohort: aged 18–41 years, BMI 19.7–25.8 kg/m^2^, N = 92	T_max_ of total and free dabigatran was prolonged from 2h to 4.5 h in the fed conditions C_max_ (ng/mL) was 144 (generic) and 147 (original) in the fasted conditions and 127 (generic) and 123 (original) in the fed conditions. However, AUC _0-_ ∞ (h·ng/mL) was 1261 (generic) 1271 (original) before the meal and 1176 (generic) and 1156 (original) after the meal			
16	GLP-1 receptor agonist	Semaglutide (van Hout et al., 2023)	Randomized, single-centre, multiple-dose, open-label, five-armed, parallel-group trial assessed alternative dosing schedules of oral semaglutide	healthy, White, Black, African American, Asian and other, male and female subjects aged 18-64 with a BMI 20–29.9 kg/m^2^; N = 156	Shorter fasting day times (from 2 to 6 h) compared to an overnight pre-dose fast, followed by a 30-min post-dose fast, resulted in significantly lower semaglutide AUC_0–24h_ and C_max_ after the 10th dose. These pharmacokinetic parameters of semaglutide were lower with a 2-h pre-dose fast and an overnight post-dose fasting period	IV (poor solubility, poor metabolism) (Bocci et al., 2022)	2 Low metabolism and a long half-life (due to low metabolism) (Bocci et al., 2022)	Semaglutide must be always taken on an empty stomach, after overnight post (8 h), with a small amount of plain water (up to 120 mL) and refrain from eating, drinking or taking medicines for at least 30 min. Splitting, crushing or chewing the tablet is not allowed since it may affect absorption of semaglutide
17	GLP-1 receptor agonist	Semaglutide (Bækdal et al., 2021)	Randomized, open-label, parallel-group, single-centre 2 trials 1. Food-effect trial, 2. Dosing conditions trail	healthy White, Black, and African American, Asian, male and female subjects aged 18-75 (mean: 55.1), with a BMI 18.5–29.9 kg/m^2^ (mean 24.8) in food-effect trial; 1.N = 78 2.N = 158	1. The food-effect trial - limited or no measurable semaglutide exposure was observed in the fed arm in comparison to the fasting arm (AUC_0–24h_ and C_max_ of semaglutide were greater by approximately 40% for the fasting versus References arm) 2. The dosing condition trial – the volume of water taken after semaglutide did not affect pharmacokinetic parameters, but the exposure increased with longer post-dose fasting			
18	SGLT-2 inhibitor	Empagliflozin (Macha et al., 2013)	Randomized, open-label, 3-way, cross-over study examined the effect of food on the pharmacokinetics of empagliflozin	healthy, White male and female subjects aged 21–51, BMI 21–29 kg/m^2^; N = 18	C_max_ was decreased when empagliflozin was taken after high-fat, high-calorie breakfast, but it was not clinically meaningful. AUC_0-_ ∞ was not changed	III (high solubility, low permeability) (Negoya et al., 2025)	1 (less than 10% of the drug is metabolised) (Hosey et al., 2015)	Administration after a high-fat meal resulted in slightly lower exposure but this was not considered clinically relevant. Empagliflozin may be administered with or without food. The tablet can be taken at any (always the same) time of the day
19	SGLT-2 inhibitor	Empagliflozin/linagliptin (Osonoi et al., 2018)	Open-label, randomized, two-sequence, crossover study examined the effect of food on bioavailability of a FDC tablet containing 25 mg empagliflozin and 5 mg linagliptin in the fed and fasted state	healthy Japanese male subjects; N = 22	AUC of empagliflozin administered in the fed state decreased by 14% and C_max_ by 25%. The values were not clinically meaningful			
20	SGLT-2 inhibitor	Empagliflozin (Li et al., 2020a)	Randomized, open-label, crossover, two-period study evaluated bioequivalence of a generic empagliflozin tablet versus a brand-and evaluate the food effects on the pharmacokinetics of empagliflozin	healthy male and female Chinese subjects N = 48 (cohorts: fasting N = 24, fed N = 24)	PK parameters including C_max_, AUC_0–t_, and AUC_0-_ ∞ and T_max_ were not affected by a high-fat meal			
21	SGLT-2 inhibitor	Empagliflozin/linagliptin (Glund et al., 2017)	Open-label, randomized, crossover study examined pharmacokinetic parameters of FDC tablet containing 25 mg empagliflozin and 5 mg linagliptin and food effect on its bioavailability	healthy volunteers; N = 42	C_max_ of empagliflozin was reduced after the meal, but overall exposure was similar to those before a meal			
22	SGLT-2 inhibitor	Empagliflozin (Chen et al., 2020)	An open-label randomized single-dose two-sequence, two-treatment, two-period crossover study compared tablet with empagliflozin to brand formulations in fed and fasted conditions	healthy Chinese male and female subjects: aged 20–38 years, BMI 19.1–26.1 kg/m^2^ N = 60 (cohorts: fasting N = 30, fed N = 30)	AUC_0–t_, AUC_0–_ ∞ , t_1/2_, and total body clearance of empagliflozin under the fed condition were similar as in fasted state T_max_ was delayed by 0.5 h in fed conditions and C_max_ decreased by 20% in these conditions Despite the small changes, food had no clinically relevant effect on the pharmacokinetics of empagliflozin	​	​	​
23	SGLT-2 inhibitor	Dapagliflozin (Kasichayanula et al., 2011)	Open-label, randomized, two-period, two-treatment crossover study evaluated the food effect on pharmacokinetic parameters of dapagliflozin	healthy (male and female) subjects aged 18–45 years, with a BMI 18–32 kg/m^2^ N = 14	C_max_ of empagliflozin was decreased by 31% and T_max_ was delayed by 1h, but AUC was not affected by administration of the drug with high-fat meal	III (Cho et al., 2021)	1 (extensive metabolism) (Kasichayanula et al., 2014)	A high-fat meal decreased mean C_max_ and increased T_max_, but did not affect AUC. It is unlikely to have a significant clinical effect. Dapagliflozin may be administered with or without food. Also, there is no clinically meaningful food effect for Dapagliflozin/Saxagliptin tablets. Because C_max_ of metformin is a smaller in the fasted state the FDC tablets are recommended to take after a meal
24	SGLT-2 inhibitor	Dapagliflozin/metformin (Chang et al., 2015)	Open-label, randomized, 4-period, 4-arm crossover 2 studies (two various doses) assessing bioequivalence and pharmacokinetics of dapagliflozin and metformin FDC tablets in healthy subjects under fed and fasting conditions	healthy White, Black Native Hawaiian/Other Pacific Islander and other male and female subjects: Study 1: aged 19–55 years, with a BMI 20.7–31.0 kg/m^2^, Study 2: aged 20–55 years, with a BMI 20.3–31.3 kg/m^2^ 1. N = 36 2. N = 36	C_max_ was lower (about 34%) and T_max_ was prolonged in both doses when the dapagliflozin/metformin tablet was taken after a light-fat meal, however AUC was only slightly reduced, T_1/2_ remained the same			
25	SGLT-2 inhibitor	Dapagliflozin/metformin (de Bruin et al., 2016)	Randomized clinical trials examining: (1) the pharmacokinetic parameters of dapagliflozin alone or on fix-dose tablet after a low-fat-meal and (2) pharmacokinetic parameters of fix-dose tablet in fasting state or after a high-fat meal	healthy predominantly White male and female subjects: Study 1: aged 18–45 years with a BMI 18–29.9 kg/m^2^, N = 120, Study 2: aged 18–55 years, with a BMI 18–30 kg/m^2^, N = 17	C_max_ of 5 mg dapagliflozin and 1000 mg metformin increased in fasted state (61,9 and 1600 ng/mL) in comparison to the fed state (43,9 and 1330 ng/mL). AUC_0-t_ was similar in both conditions			
26	SGLT-2 inhibitor	Dapagliflozin/metformin or saxagliptin (Tang et al., 2019)	Randomized, open-label, single-dose, single-centre crossover study evaluating the pharmacokinetic parameters in the fasted and fed conditions of fix-combination drug products including dapagliflozin, metformin and saxagliptin in various doses	healthy White, Black or African American, Asian male and female subjects; Cohort 1: aged 18–55 years, BMI 18–32 kg/m^2^ Cohort 2: aged 18–55 years, BMI 18–32 kg/m^2^; N = 84 (Cohort 1: N = 42 Cohort 2: N = 42)	T_max_ of dapagliflozin was delayed in the fed state versus the fasted state (2.00 vs. 0.98 h, respectively) and C_max_ value was lowered by 38% after a meal, but the values of AUC were within 80%–125% limit	​	​	​
27	SGLT-2 inhibitor	Dapagliflozin/metformin (Vakkalagadda et al., 2016)	Randomized open-label, single-dose crossover study examining the bioequivalence of FDC tablet (dapagliflozin/metformin) and the single drugs and the food effect on these formulations	healthy White, Black or Asian male and female subjects aged 20–49 years (mean: 34.0), with a BMI 19.7–29.8 kg/m^2^ (mean 25.63); N = 72	T_max_ of dapagliflozin was prolonged in the presence of food (3 h versus 1 h) and C_max_ was reduced from 35% to 50% in the presence of food. However, the AUC was not significantly changed			
28	SGLT-2 inhibitor	Dapagliflozin/metformin (Boulton et al., 2016)	An open-label, randomized, 2-way crossover, 4-arm study assessing the bioequivalence of bioequivalence of prolong acting FDC tablet (dapagliflozin/met-formin) and individual components drugs in fed and fasted state	healthy Brazilian male and female subjects aged 18–55 years, BMI 18.5–28.5 kg/m^2^; N = 129 Arm 1: N = 34 Arm 2: N = 29 Arm 3: N = 34 Arm 4: N = 32	1. Dapagliflozin/metformin 5/500 mg XR tablet: Dapagliflozin C_max_ was higher and T_max_ was shorter in the fasted state in comparison to the fed state for both FDC tablet and the single drugs, but AUCs were consistent under these conditions 2. Dapagliflozin/metformin 10/1000 mg XR tablet. The parameters regarding dapagliflozin were similar as previously. Only C_max_ of metformin was significantly smaller in fasted conditions			
29	SGLT-2 inhibitor	Canagliflozin (Devineni et al., 2015)	Randomized crossover two studies examining pharmacokinetic parameters of canagliflozin used under fed and fasted conditions	Healthy predominantly White male and female subjects aged 18–55 years with a BMI 18–30 kg/m^2^ 1. N = 24 2. N = 24	C_max_ and AUC ∞ fed/fasted geometric mean ratios were 100.51 ng/mL and 108.09 ng h/mL and the parameters were within bioequivalence limits (80%–125%) Additionally, administration of canagliflozin on the empty stomach reduced intestinal glucose absorption over the first 2 h after a meal.	II (low solubility, high permeability) (Devineni et al., 2013)	2 (extensive metabolism) (Hosey et al., 2015)	No clinically relevant food effects on canagliflozin exposure parameters were observed, however administration of canagliflozin before a meal reduced intestinal glucose absorption over the first 2 h after a meal. It is recommended to take the drug before the first meal of the day. However, if canagliflozin is taken in one tablet with metformin it is recommended to take after the food to avoid GI disturbances
30	SGLT-2 inhibitor	Canagliflozin/metformin (Murphy et al., 2015)	Randomized, open-label, single-dose, 2-period, 2-sequence crossover study assessing the effect on food on pharmacokinetic parameters of fixed-dose combination (FDC) tablet containing canagliflozin and metformin	healthy subjects; N = 24	The pharmacokinetic parameters of canagliflozin as C_max_ and AUC of 150/1000 mg FDC tablet containing canagliflozin/metformin administered in fed and fasted conditions were bioequivalence limit (80%–125%). The C_max_ of metformin decreased by 16% in the fed conditions, however it was not clinically meaningful			
31	SGLT-2 inhibitor	Ertugliflozin (Raje et al., 2018)	A two-period study design was applied to determine the absolute oral bioavailability (F) and fraction absorbed (F_a_) of ertugliflozin with^14^ C microtracer dosing	healthy White male subjects aged 25–54 years with a BMI 20.3–29.7 kg/m^2^; N = 8	Under fasted conditions oral bioavailability of ertugliflozin was complete and the value was about 100%	I (high solubility, high permeability, under fasting condition rapid and 100% absorption) (Bocci et al., 2022; Fediuk et al., 2020)	1 (high solubility, primary metabolized) (Bocci et al., 2022)	Ertugliflozin is well absorbed from the gastrointestinal tract and can be taken regardless of meal. If the patient has trouble swallowing, the tablet can be broken or crushed because it’s immediate-release (information from the leaflet)
32	SGLT-2 inhibitor	Ertugliflozin (Li et al., 2021)	Randomized, double-blind study in healthy Japanese subjects and open-label study in Western subjects assessed pharmacokinetics and pharmacodynamics of ertugliflozin	healthy Japanese and Western (Asian, Black and White) male and female (non–childbearing) subjects aged 27–54 years with a BMI 17.7–28.3 kg/m^2^; N = 24	Under fasted conditions the median T_max_ of ertugliflozin was 1.00–1.50 h and under fed conditions was delayed to 2.50			
33	SGLT-2 inhibitor	Ertugliflozin/metformin or sitagliptin (Sahasrabudhe et al., 2019)	Randomized, 2-period, single-dose, crossover studies (three) assessing the effect of a standard high-fat breakfast on the pharmacokinetics of ertugliflozin alone and the fixed-dose combination (ertugliflozin/metformin, ertugliflozin/sitagliptin) formulations	healthy White, Black and other male and female subjects, Ertugliflozin study: age 25–53 years with a BMI 22.2–29.2 kg/m^2^. Ertugliflozin/Sitagliptin study: age 20–54 years, BMI 20.3–29.6 kg/m^2^. Ertugliflozin/Metformin study: age 20–52 years, BMI 19.3–28.6 kg/m^2^ N = 52 (14 subjects in each study)	T_max_ for ertugliflozin (alone or in combination) in the fasted conditions was 1.0–1.5h, and in the fed conditions was prolonged to 2.0–2.5 h. A high-fat meat decreased C_max_ by 29% (alone), by 30% when administered as the ertugliflozin/sitagliptin FDC, and by 41% when administered as the ertugliflozin/metformin FDC tablet. However, the AUC was similar in both conditions			
34	Hypolipemic drugs	Bempedoic acid (Amore et al., 2023)	Phase 1, single- and multiple ascending dose, and food effect assessing the pharmacokinetics of bempedoic acid	healthy Japanese, Chinese, and Western male and female (not pregnant or lactating) subjects aged 18–60 years with a BMI 18.0–40.0 kg/m^2^patients with mild hyperlipidemia; N = 83 (healthy subjects) N = 24 (patients)	C_max_ of bempedoic acid was decreased by 13% in the presence with food, however the AUC exposure was not significantly different between fed and fasted status. In general, the PK parameters after a high-fat meal were similar to fasted state and the slight differences were not significant	II (low water solubility in acidic environment and high permeability, well absorbed in the intestine (Ferri et al., 2024)	2 (extensive metabolism and low solubility) (Bocci et al., 2022; Ferri et al., 2024)	Bempedoic acid may be taken without regard to meal
35	Statin	Pitavastatin (Shang et al., 2016)	Open label, randomized, crossover clinical trial investigating the effect of food on the pharmacokinetics two formulations of pitavastatin (the original and the generic) of the	healthy male Chinese subjects aged 20–30 years with a BMI 19.38–23.05 kg/m^2^; N = 24	The AUC_0–48h_ decreased to 87.69% (generic) and 83.7% (branded) and AUC_0–_ ∞ decreased to 87.50% (generic) and 84.6% (branded) in the presence of food. C_max_ was also decreased to 45% (generic) and 50,4% (branded) and T_max_ was extended by 2.4 folds. However, food did not affect the plasma concentrations of pitavastatin in the fed and fasted groups at the last four time point were the same	II (Wang et al., 2022)	4 (Hosey et al., 2015)	Fat breakfast may reduce Cmax. When taken in the morning it is better to take it on the empty stomach, however according to the manufacturer the drug can be taken regardless of a meal. It is rather not affected by grapefruit juice during metabolism
36	​	Pitavastatin (Ando et al., 2005)	Open, randomized, four-phase crossover design with the intervals of 2 weeks study comparing the effects of grapefruit juice on the pharmacokinetics of pitavastatin and atorvastatin	healthy male Japanese subjects, aged 23–34 years; N = 8	The mean AUC_0–24_ of pitavastatin acid was increased by only 13% after grapefruit juice (in the case of atorvastatin it was 83%). Moreover, the C_max_ remained unchanged			

## 3 Results

### 3.1 Main findings

**FIGURE 2:**
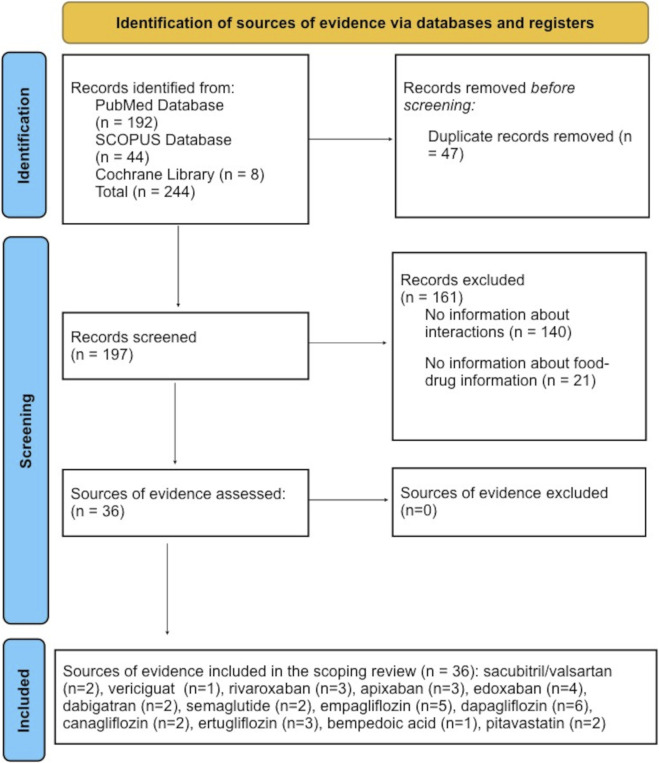
PRISMA-ScR flow diagram illustrating the study selection process for the scoping review, adapted from the PRISMA-ScR guidelines (Tricco et al., 2018). The work is Licensed under CC BY 4.0.

A total of 244 records were identified, and 47 duplicates were removed. We initially yielded a total of 197 records which fulfilled the research strategy criteria. Following a thorough screening process–including abstract review and full-text assessment where necessary – 36 sources of evidence were selected for inclusion in this PRISMA-ScR. Figure 2 presents the PRISMA-ScR flow diagram that illustrates the selection process. Thirteen agents across cardiology and diabetology were represented, most frequently SGLT2 inhibitors and DOACs. Table 1 summarizes the search results, including the source of evidence, pharmacological class, active substance, study design, population, main outcomes, BCS and BDDCS classifications, information from the leaflet and FDA recommendations, and recommendations regarding the appropriate use of the drug.

The studies included between 8 and 255 participants, with most studies involving more than 30 participants. Most studies evaluated food effects on single-agent formulations. However, several investigations examined the influence of food on fixed-dose combination (FDC) tablet, including SGLT2 inhibitors combined with metformin (Boulton et al., 2016; de Bruin et al., 2016; Chang et al., 2015; Murphy et al., 2015; Sahasrabudhe et al., 2019; Tang et al., 2019; Vakkalagadda et al., 2016), linagliptin (Glund et al., 2017; Osonoi et al., 2018), saxagliptin (Tang et al., 2019) and sitagliptin (Sahasrabudhe et al., 2019). Four studies examined the administration the drug in crushed form (Duchin et al., 2018; Moore et al., 2014; Song et al., 2016; 2015), a further six examined various doses of drugs (Akahori et al., 2017; Boettcher et al., 2021; Chang et al., 2015; Kubitza et al., 2006; Stampfuss et al., 2013; Tang et al., 2019), and three studies examined the influence of various diet types on pharmacokinetic parameters (Ayalasomayajula et al., 2016; de Bruin et al., 2016; Kubitza et al., 2006).

In most studies, AUC was comparable between fed and fasted conditions. When differences were observed, they were typically small and seldom resulted in clinically meaningful consequences. Notable exceptions included vericiguat and higher doses of rivaroxaban, which showed improved drug exposure when taken with food. This suggests that it is appropriate for patients to take these drugs with a meal (Boettcher et al., 2021; Kubitza et al., 2006; Stampfuss et al., 2013). On the contrary, when considering oral semaglutide, the ingestion of food markedly reduced bioavailability and the drug was recommended to be taken only on an empty stomach (Bækdal et al., 2021; van Hout et al., 2023). Another study examining pitavastatin, showed a decrease in total exposure in fed conditions, however the drug can be taken with a meal (Shang et al., 2016).

### 3.2 Cross-drug synthesis of food-drug interactions across pharmacological classes

The included drugs were grouped into major pharmacological classes–direct oral anticoagulants (DOACs) and sodium–glucose cotransporter 2 (SGLT2) inhibitors–as well as individual agents that do not form homogeneous classes within the scope of this review, including oral semaglutide (the only orally administered glucagon-like peptide-1 receptor agonist), sacubitril/valsartan, vericiguat, pitavastatin, and bempedoic acid.

Across these groups, distinct patterns in food-related pharmacokinetic effects were observed. DOACs generally exhibited limited or dose-dependent food effects, with most studies reporting comparable systemic exposure in under fed and fasted conditions. An exception was rivaroxaban, for which food intake influenced bioavailability in a dose-dependent manner. In addition, studies demonstrated that tablets containing direct factor Xa inhibitors may be administered in crushed form and mixed with food without clinically relevant changes in exposure.

SGLT2 inhibitors showed largely consistent pharmacokinetic profiles, irrespective of food intake. The majority of studies reported minimal or no clinically relevant differences in AUC or C_max_ between fed and fasted states, and findings were consistent across both single-agent formulations and fixed-dose combination.

Among medicines used for elevated LDL cholesterol levels, bempedoic acid demonstrated comparable systemic exposure under fed and fasted conditions, indicating that administration is not dependent on meal timing. Pitavastatin exhibited a higher C_max_ when under fasting conditions. Vericiguat, a drug used in the management of heart failure, showed improved pharmacokinetic parameters when administered with food.

In contrast, glucagon-like peptide-1 receptor agonists, represented in this review by oral semaglutide, consistently exhibited marked reductions in systemic exposure when administered with food, indicating pronounced sensitivity to fed conditions.

Overall, these class-specific patterns indicate that the magnitude and direction of food-drug interactions are not uniform across pharmacological groups and are influenced by underlying biopharmaceutical properties and formulation-related factors.

### 3.3 Integration of food effects with BCS and BDDCS classification and mechanistic explanations for food effects

To provide a more comprehensive characterization of the analyzed medicines, BCS and BDDCS categories were assigned and are presented in Table 1. Inclusion of BCS and BDDCS categories facilitates a mechanistic interpretation of the observed pharmacokinetic findings and aids in the anticipation of potential clinical implications, particularly in the context of concomitant drug administration.

From a mechanistic perspective, highly soluble drugs classified as BCS Class I or III generally show limited sensitivity to food intake, which is consistent with their high solubility and/or permeability, reducing dissolution-dependent variability in absorption. Class I drugs, such as Sacubitril and Ertugliflozin, typically exhibit minimal food effect on overall exposure. In contrast, Class III drugs, including Apixaban, Empagliflozin, and Dapagliflozin, are permeability-limited; therefore, their bioavailability may decrease in the presence of a high-fat meal due to interactions with intestinal transport processes. In comparison, BCS Class II drugs often demonstrate increased bioavailability with food, likely due to enhanced solubilization in the presence of dietary lipids, as observed for rivaroxaban. Finally, the absorption of Class IV drugs is highly variable and strongly dependent on formulation, as both solubility and permeability are limiting factors; this variability can be seen with semaglutide.

The analysis of the BDDCS classification of the studied drugs indicates that five compounds belong to class 2, which is characterized by extensive metabolism, low solubility but high permeability. This category includes vericiguat, rivaroxaban, canagliflozin, semaglutide and bempedoic acid and their exposure depends on food intake or formulation.

Drugs from class 1 as, e.g., sacubitril, apixaban, flozines apart from canagloflozin represents compounds where the AUC is not affected by a high-fat meal. In contrast, several agents, including valsartan, edoxaban, dabigatran, and pitavastatin, are classified as BDDCS class 4, characterized by limited metabolism and potential involvement of transporters in their absorption and disposition.

## 4 Discussion

Patients with CVD and T2DM constitute a very large group of patients in clinical practice (Marx et al., 2023). Early diagnosis enables the prompt initiation of effective therapy. In most patients suffering from both diseases, four to five drugs are needed to attain therapeutic efficacy which substantially increases the risk of drug-drug interactions (Marx et al., 2023). Many drugs traditionally used in cardiology and diabetology are also subject to FDIs. These are well known and therefore preventable, such as the impact of food products containing large amounts of vitamin K on the effectiveness of warfarin and acenocoumarol (Van Gelder et al., 2024; Nutescu et al., 2006).

In recent years, several novel pharmacological classes have been incorporated into routine clinical practice and now play a central role in the management of CVD and T2DM (Marx et al., 2023). Evidence describing the magnitude and clinical relevance of FDI’s for these agents has gradually emerged. Within the scope of the present review, 36 studies met the predefined eligibility criteria and formed the basis for the following synthesis (Table 1). In most of the included studies, investigators primarily focused on the influence of food on fundamental pharmacokinetic parameters reflecting systemic exposure, such as C_max,_ T_max_, and AUC. A smaller subset of studies additionally explored the role of meal composition or caloric content in modifying these outcomes. Overall, for most contemporary agents used in the treatment of CVD and T2DM, food intake did not translate into clinically meaningful alterations in therapeutic effectiveness. Nevertheless, variability in pharmacokinetic behavior was observed. Depending on the compound, food could increase or decrease bioavailability, although in most instances total drug exposure remained largely unaffected. In select cases, particular consideration of pharmacodynamic consequences was warranted (Table 1).

### 4.1 Novel drugs for heart failure and/or diabetes type 2

For many years, heart failure (HF) has remained one of the most difficult challenges in modern cardiology (McDonagh et al., 2022). Despite the significant progress that has been made in the diagnosis and treatment of this disease in recent years, the prognosis is still very serious (McDonagh et al., 2022). Recently, drugs that have significantly reduced the mortality and morbidity of this disease have been widely implemented into clinical practice, including: ARNI, SGLT2 inhibitors (mostly empagliflozin, dapagliflozin) and vericiguat (McDonagh et al., 2022; Metra and Teerlink, 2017; Zhang et al., 2023). In addition, many groups of drugs have been introduced that improve the prognosis of patients suffering from HF or other CVD and T2DM, particularly SGLT2 inhibitors and GLP-1 receptor agonists.

#### 4.1.1 Angiotensin receptor neprilysin inhibitor: sacubitril/Valsartan

ARNI is recommended in the treatment of HF with reduced ejection fraction (HFrEF) (class Ia) and it may be considered in the treatment of HF with mildly reduced ejection fraction (HFmrEF) (class IIa) (McDonagh et al., 2022). Valsartan/sacubitril is administered orally, twice daily, as recommended, regardless of meals (McDonagh et al., 2022). According to the data from the summary of product characteristics, meals have no clinically significant effect on the AUC of both substances. It should be emphasized that sacubitril is a prodrug, therefore food has a limited effect on the concentration of the active metabolite (LBQ657, sacubitrilat) in the blood. The two analysed studies showed food can reduce the C_max_ and AUC of valsartan and sacubitril, but has only a negligible effect on systemic exposure of sacubitrilat (Akahori et al., 2017; Ayalasomayajula et al., 2016). Notably, due to the possibility of side effects such as deterioration of kidney function and hyperkalaemia when using valsartan/sacubitril, excessive consumption of products rich in potassium should be limited (McDonagh et al., 2022). Moreover, the diet supplements including potassium or salt substitutes should be avoided, or their usage discuss with the physician. Due to the minimal metabolism of sacubitril and valsartan via CYP450 enzymes, the concomitant administration of food products and drugs affecting CYP450 enzymes is not expected to affect the pharmacokinetics of this drug, which undoubtedly reduces the risk not only of food-drug, but also drug-drug interactions. Since ARNI consists also of valsartan, it is worth mentioning that valsartan can also be safely used with or without food, which was confirmed by Sunkara et al. who evaluated the FDC tablet with amlodipine and/or hydrochlorothiazide (Sunkara et al., 2014). Additionally, age, sex, and ethnicity were not found to influence the pharmacokinetics of sacubitril/valsartan. However, when comparing patients with heart failure to healthy individuals, systemic exposure was higher in the patient group (Ayalasomayajula et al., 2017). In summary, due to the lack of reported clinically meaningful impact of food on the pharmacokinetic parameters of ARNI, the drug should be used twice a day with or without food in patients with HF without affecting its therapeutic effectiveness.

#### 4.1.2 Sodium-glucose cotransporter 2 inhibitors: empagliflozin, dapagliflozin, ertugliflozin, canagliflozin

The introduction of SGLT2 inhibitors in the treatment of patients with CVD and T2DM has proven to be a significant breakthrough in cardio-diabetology (Marx et al., 2023; McDonagh et al., 2023; McDonagh et al., 2022). Beyond promoting urinary glucose excretion, these agents exert pleiotropic actions that translate into cardioprotective and nephroprotective benefits (McDonagh et al., 2023; McDonagh et al., 2022). Randomized trials established empagliflozin and dapagliflozin as key therapies not only for T2DM but also for heart failure with reduced ejection fraction (HFrEF), with proven prognostic benefit (McDonagh et al., 2021; McMurray et al., 2019; Packer et al., 2020).

Importantly, they were also the first drug class shown to improve outcomes in heart failure with preserved ejection fraction (HFpEF) (McDonagh et al., 2023; Solomon et al., 2022). Current recommendations support their use in patients with T2DM and chronic coronary syndrome irrespective of HbA1c levels or background glucose-lowering therapy (Vrints et al., 2024).

SGLT2 inhibitors can generally be administered with or without food and are available as an FDC tablet with metformin or DPP-4 inhibitors. Across the 16 publications included in this analysis, food had no clinically meaningful impact on therapeutic effectiveness. All flozins, apart from canagliflozin, belong to the first BDDCS class, what means that the effect of food is minimal.

For empagliflozin, most studies reported a reduction in C_max_ with largely unchanged AUC in fed conditions, particularly after the ingestion of high-fat, high-calorie meals; these differences were not considered clinically relevant (Chen et al., 2020; Glund et al., 2017; Macha et al., 2013). Similar conclusions apply to the empagliflozin/linagliptin FDC, where modest decreases in exposure did not translate into clinical relevance (Glund et al., 2017; Osonoi et al., 2018). Consistent findings were reported for dapagliflozin: post-meal administration may lower C_max_ and prolong T_max_ but does not materially alter AUC, whether the drug is given alone or with metformin or saxagliptin (Boulton et al., 2016; de Bruin et al., 2016; Chang et al., 2015; Kasichayanula et al., 2011; Tang et al., 2019; Vakkalagadda et al., 2016). Likewise, food does not meaningfully influence the bioavailability of canagliflozin or ertugliflozin (Devineni et al., 2015; Li et al., 2021; Murphy et al., 2015; Raje et al., 2018). Some practical considerations have been proposed. Canagliflozin taken before the first meal may enhance control of postprandial glycaemia (Devineni et al., 2015). When combined with metformin, administration with meals is often advised to mitigate gastrointestinal intolerance (Murphy et al., 2015; Sahasrabudhe et al., 2019).

In summary, although food may modestly reduce C_max_, it does not significantly affect overall exposure or clinical efficacy. Therefore, SGLT2 inhibitors can generally be used irrespective of meals, while combination products containing metformin are preferably taken with food to improve gastrointestinal tolerability.

#### 4.1.3 Glucagon-like peptide 1 receptor agonists

Most GLP-1 receptor agonists are administered subcutaneously, so the risk of FDIs is low. The only currently available drug from this group also used orally is semaglutide, which is formulated with sodium N-[8-(2-hydroxybenzoyl)aminocaprylate] (SNAC) which buffers the environment protecting the active substance from enzymatic degradation and enhances its transcellular absorption from the gastrointestinal tract (Buckley et al., 2018).

Despite the effect on the stomach, semaglutide does not significantly affect the overall exposure of other drugs, including those with narrow therapeutic windows (Calvarysky et al., 2024). It should be noted that semaglutide has a multifaceted effect not only in regulating appetite and glucose levels, but also in influencing cardiovascular system function. According to current guidelines, it is a drug of choice in addition to SGLT2 inhibitors in patients with T2DM and CVD (Marx et al., 2023). Moreover, recent studies have indicated that oral formulations of semaglutide (50mg/24 h) in adults with increased body mass without T2DM lead to a remarkable and clinically meaningful decrease in bodyweight compared with placebo (Knop et al., 2023). According to the product characteristics, the drug is absorbed primarily in the stomach, and its absorption is reduced if taken with food or large amounts of water, therefore prolonged fasting after dosing increases absorption. Also, in the leaflet is a recommendation to not split, crush or chew the tablet, as it may affect absorption of the active substance. Baekdal TA et al. showed that food reduces the AUC_0–24h_ and C_max_ of semaglutide by about 40%, therefore it is recommended to administer the drug with a small volume of plain water (up to 120 mL) and to remain on an empty stomach for at least 30 min after dosing (Bækdal et al., 2021). Van Hout M et al. confirmed these findings, and highlighted that shorter fasting day times (2–6 h) resulted in lower semaglutide exposure in comparison to overnight pre-dose fasting, indicating that the oral form of this drug should be administered on an empty stomach and that food, liquids and other medicines should be taken at least 30 min after dosing (van Hout et al., 2023).

#### 4.1.4 Soluble guanylate cyclase stimulators: Vericiguat

Vericiguat has been shown to reduce cardiovascular death or hospitalization in patients with heart failure with reduced ejection fraction (HFrEF) and may be considered in those who remain symptomatic despite guideline-directed therapy (Armstrong et al., 2020; McDonagh et al., 2022). In contrast to SGLT2 inhibitors, food intake has a clinically relevant impact on its pharmacokinetic profile. When administered with meals, vericiguat achieves an oral bioavailability of approximately 93%. A high-fat, high-calorie meal decreases pharmacokinetic variability and increases exposure by about 19% and C_max_ by around 9%. Moreover, vericiguat is classified as BCS Class 2 and BDDCS Class II, indicating low solubility and high permeability with extensive metabolism; therefore, food may enhance its absorption and bioavailability by improving solubilization in the gastrointestinal tract. The tablet is typically taken once daily at approximately the same time, and according to the manufacturer it may be crushed for patients who have difficulty swallowing. Consequently, intake with food is recommended to ensure more predictable and stable drug exposure.

### 4.2 Novel hypolipemic drugs

#### 4.2.1 Bempedoic acid

Bempedoic acid is used in patients who are statin intolerant and fail to achieve lipid targets on ezetimibe, or in those who remain above their goal, despite maximally. It has similar actions on the reduction of cardiovascular risk as statins, but it is associated with a smaller risk of muscle-related adverse effects (Lincoff et al., 2024). From a pharmacokinetic perspective, bempedoic acid is well absorbed, demonstrates high oral bioavailability, and is generally well tolerated at the gastrointestinal level (Ruscica et al., 2022). Available evidence indicates that it can be administered irrespective of meals. In a study by Amore et al., the administration of this drug whilst in a fed state resulted in a reduction in C_max_ without a meaningful effect on overall exposure, supporting the absence of clinically relevant food interactions (Amore et al., 2023).

#### 4.2.2 Pitavastatin

Statins are the foundational drugs used to treat dyslipidemia and prevent CVD. For over 30 years of their use, their effectiveness in lowering LDL cholesterol levels and reducing the risk of cardiovascular events has been confirmed. Statins are generally considered safe, however, in some cases, the concomitant intake of food, especially grapefruit juice, may potentiate the incidence of adverse drug reactions. This is apparent especially in older statins, as, e.g., simvastatin and atorvastatin (Lilja et al., 1999; Lilja et al., 1998). Pitavastatin is one of the newest statins, registered in 2009, which has recently experienced a renaissance in cardiac pharmacotherapy. Pitavastatin stands out from other statins due to its favourable safety profile, in particular the lower risk of inducing myalgia and myopathy and a less severe effect on blood glucose levels. It is also one of the few statins, that is not metabolized by CYP450, which significantly decreases the risk of drug-drug and FDIs, particularly foods that affect CYP3A4 activity (Ando et al., 2005). Shang et al. reported that in the Chinese population, food intake (a high-fat breakfast) was shown to markedly affect the pharmacokinetic profile of pitavastatin, with a significant reduction in C_max_ and modest lowering of the AUC compared with fasting conditions, consistent with previously published data in healthy subjects (Shang et al., 2016). In contrast, regulatory and labelling data from the FDA indicate that while a high-fat meal decreases pitavastatin C_max_ by about 43%, it does not significantly reduce the overall AUC, suggesting limited impact on total systemic exposure. Taken together, these findings suggest that although prandial state can substantially alter the rate of absorption and peak levels of pitavastatin, the effect on overall exposure may be modest and not uniformly observed across all studies. Nevertheless, further consideration of whether pitavastatin is administered with or without food may be warranted in clinical settings to minimize variability in pharmacokinetic exposure.

### 4.3 Novel anticoagulants: direct oral anticoagulants

DOACs in many countries are the most common used anticoagulants for most indications (Hindley et al., 2023). Because of their properties they are recommended in patients with atrial fibrillation to prevent thromboembolic complications including stroke (Van Gelder et al., 2024; Hindley et al., 2023).

Due to their favourable efficacy and safety profile, DOACs have largely replaced the once widely used vitamin K antagonists (VKAs), such as warfarin or acenocoumarol, which are currently dedicated mainly to patients with valvular atrial fibrillation, mechanical valve implantation and patients with severe renal failure (GFR<15 mL/min/1.73 m2) (Van Gelder et al., 2024). VKAs have a narrow therapeutic window and are associated with a high risk of interactions with medicines, food and food supplements, which can potentiate or decrease the anticoagulant effect (Di Minno et al., 2017). Their adverse reactions and low safety profile may also discourage patients from continuing therapy (Hylek et al., 2007). Although, DOACs are safer, analyses of single clinical cases indicate that it is important to limit the consumption of grapefruit juice and St. John’s wort in patients whilst being concurrently treated with DOACs due to dangerous interactions (Walenga and Adiguzel, 2010).

However, there is little is known about FDIs. Most authors suggested that food may have a positive effect on rivaroxaban and edoxaban pharmacokinetics (Kubitza et al., 2006; Liu et al., 2022; Mendell et al., 2011; Stampfuss et al., 2013). The increased bioavailability is especially visible when higher doses are administered (20 mg of rivaroxaban) (Stampfuss et al., 2013). Kubitza D et al. showed that rivaroxaban, especially in high doses, prolonged prothrombin time when used in fed state (PT prolongation by 53% in the fasted state, and by 83% after food). Despite the improvement in pharmacodynamic parameters, the type of diet consumed did not have a significant effect on the change in pharmacokinetic parameters (Kubitza et al., 2006). Chen X et al. suggested that food may play an important role in the formation of the active metabolite of edoxaban, since its postprandial exposure was slightly lower. Nevertheless, this drug can be administered regardless of meals (Chen et al., 2017). With respect to apixaban, Frost C et al. did not find any differences in C_max_ and AUC between fed and fasted conditions and furthermore, various kinds of foods did not affect pharmacodynamic parameters. Therefore, it can be concluded that apixaban can be administered regardless of meals (Frost et al., 2013).

The only oral representative for factor IIa inhibitors, dabigatran etexilate, can be safety administered with food. Studies found that food did not change the extent of drug absorption, however caused up to a two-fold prolongation of T_max_ (Li et al., 2020b; Stangier et al., 2005).

It should be emphasized that in fed state, T_max_ for DOACs can be delayed, which may have important clinical implications (Frost et al., 2013; Kubitza et al., 2006; Li et al., 2020b; Stampfuss et al., 2013). Due to their rapid onset of action, DOACs are not used concurrently with heparins. Therefore the clinical relevance of food-induced delays in T_max_ remains uncertain. This may be particularly important in high-risk settings, such as the early perioperative period, where further studies are needed to determine whether such pharmacokinetic changes have meaningful effects on thromboembolic outcomes.

An important aspect of safe pharmacotherapy, alongside meal timing, is the method of drug administration. In general, tablets should not be crushed or split unless explicitly indicated, as this may irritate the esophagus, modify absorption, or result in unintended immediate release of the active substance, particularly in prolonged- or extended-release formulations. Similarly, capsule contents should not be emptied due to the risk of altered pharmacokinetics. However, in specific clinical scenarios–such administration via a percutaneous endoscopic gastrostomy (PEG) or nasogastric (NG) tube, or in patients with dysphagia, modification of the dosage form may be unavoidable. Evidence from several studies indicates that edoxaban, apixaban, and rivaroxaban can be given as crushed tablets, either mixed with applesauce or suspended in water for NG tube administration, without clinically meaningful changes in exposure (Duchin et al., 2018; Moore et al., 2014; Song et al., 2016; 2015). Furthermore, according to prescribing information, ertugliflozin and vericiguat may also be crushed and administered safely. Overall, while many cardiovascular agents can be used irrespective of meals, practical considerations related to swallowing ability and alternative routes of delivery may ultimately determine the optimal method of administration.

## 5 Study limitations

Although formal risk-of-bias assessment is not required in scoping reviews, several factors related to study design should be considered when interpreting the findings. The studies included in this review varied in sample size, ranging from 8 to 255 participants, with most studies including more than 30 subjects. Two studies involved smaller cohorts. One investigated ertugliflozin; however, its results were consistent with findings from other studies with larger populations. The second examined the effect of grapefruit juice on the pharmacokinetics of pitavastatin, particularly the area under the curve (AUC), and supported existing evidence indicating that pitavastatin metabolism is not dependent on CYP3A4. Moreover, for most drugs included in the review, between two and six studies were available. The only exception was vericiguat, for which a single study was identified; however, it included the largest sample size (255 participants), which may strengthen the reliability of the reported findings.

Whilst the review was focused on identifying the FDIs of novel drugs used in cardiologic and diabetic patients, most of the studies included in the literature review were conducted in groups of healthy volunteers, which may impact on the generalizability of the findings. There is a distinct lack of randomized trials published on FDIs in the CVD and/or T2DM patient population, and it is generally assumed that the pharmacokinetic and pharmacodynamic properties of drugs used in these patients correspond to the information contained in the summary of product characteristics.

We found and included only 36 research papers. Therefore, it should be emphasized that there are significant limitations in extrapolating interpretations of fed/fasted pharmacokinetic data obtained from healthy subjects to patients with cardiovascular diseases and diabetes mellitus. A particularly important group comprises patients with HF, which is often accompanied by absorption disorders resulting primarily from intestinal mucosal ischemia, venous congestion in the gastrointestinal tract, and chronic inflammation; these factors may lead to alterations in the pharmacokinetic parameters of many drugs. Moreover, in patients with long-standing T2DM, especially when inadequately controlled, gastroparesis may occur as one of the complications of diabetic polyneuropathy. This condition leads mainly to delayed gastric emptying, which often results in the incomplete absorption of many drugs from the small intestine. Finally, it should be remembered that the majority of patients with CVD and T2DM are elderly; a patient group with pre-existing comorbidities that alter the pharmacokinetics of many drugs. These include, among others, an increase in gastric pH, slowed gastric emptying, reduced body water and albumin content, decreased hepatic enzyme function, and reduced renal clearance. In all elderly patients, especially those with cachexia and frailty, the dosage of some drugs should be adjusted to the body weight, renal and hepatic function. In addition, many elderly patients have multiple comorbidities resulting in polypharmacy, which significantly increases the risk of dangerous interactions–not only with food but also drug-drug interactions–as well as the associated adverse effects and hospitalizations.

The review did not consider the results of clinical trials on interactions between food products and herbs, limiting the generalizability of findings.

Despite the above limitations, the presented review may provide an important starting point for a discussion on FDIs in patients with CVD and/or T2DM. Further randomized clinical trials are needed to assess the clinical significance of the effect of food on drug pharmacokinetics in the cardiac-diabetic population particularly burdened with comorbidities.

## 6 Conclusion

In conclusion, most drugs used in modern therapeutic regimens for CVD and T2DM can be taken with or without food. Although the influence of food on some pharmacokinetic parameters has been confirmed, in the majority of cases, its adverse effects on bioavailability was not observed. Therefore it is considered insignificant from a clinical point of view.

However, the evidence highlighted that food could increase the bioavailability of certain drugs, such as vericiguat and rivaroxaban therefore necessitating concurrent administration with a meal. In contrast, the bioavailability of orally taken semaglutide was significantly reduced in the presence of food, requiring the drug to be taken on an empty stomach under strict restrictions.

Most evidence derives from studies in healthy volunteers, highlighting the need for further research in patients with CVD and T2DM. Overall, understanding optimal drug administration, including meal timing–can directly enhance adherence, safety, and therapeutic effectiveness. Clinicians should integrate these considerations into practice to maximize the benefits of novel cardiovascular and antidiabetic therapies.
